# Co‐Occurring Sister Taxa of Mountain Butterflies Exhibit Distinct Cuticular Hydrocarbon Profiles

**DOI:** 10.1002/ece3.72027

**Published:** 2025-09-03

**Authors:** Irena Kleckova, Mary Veronica Clancy, Camille Cornet, Nadir Alvarez, Pável Matos‐Maraví, Kay Lucek

**Affiliations:** ^1^ Biology Centre CAS Institute of Entomology České Budějovice Czech Republic; ^2^ Fundamental and Applied Research in Chemical Ecology, Institute of Biology University of Neuchâtel Neuchâtel Switzerland; ^3^ Biodiversity Genomics Laboratory, Institute of Biology University of Neuchâtel Neuchâtel Switzerland; ^4^ Naturéum—State Museum of Natural Sciences Lausanne Switzerland; ^5^ Department of Ecology and Evolution University of Lausanne Lausanne Switzerland

**Keywords:** CHCs, contact pheromones, Lepidoptera, secondary contact, semiochemicals

## Abstract

Invisible to human perception, differentiation in chemical traits such as insects cuticular hydrocarbons (CHCs) might contribute to speciation. The species‐rich mountain butterfly genus *Erebia* represents a well‐established model for studying speciation because closely related taxa form stable secondary contact zones. However, to which degree these taxa would also differ in their chemical composition of the cuticle has remained unexplored. We compared CHCs of males and females from four locally sympatric or parapatric sister taxa pairs with varying levels of gene flow. Rarely hybridizing taxa pairs (*E. cassioides*—*E. tyndarus*, *E. euryale*—*E. ligea*) exhibited significant CHC differentiation at both interspecific and intersexual levels. Conversely, taxa pairs with no prior contact (*E. melampus*—*E. sudetica*) or frequent ongoing hybridization in their contact zones (*E. euryale adyte*—*E. e. isarica*) showed limited CHC differentiation. Our findings suggest that differentiation in CHC profiles scales with among‐species gene flow. Although it remains unclear whether CHCs are involved in mate recognition in *Erebia*, the observed differentiation could play a role in reproductive isolation, particularly under environmental changes that promote novel interspecific interactions. Future research should explore the role of CHC divergence across hybrid zone gradients and pinpoint the genomic regions underlying CHC synthesis and perception.

## Graphical abstract

Effect sizes of variance explained by redundancy analyses (RDAs) comparing cuticular hydrocarbon profiles of related species and subspecies pairs of *Erebia* butterflies. Variability attributed to taxon was highest in *E. cassioides* and *E. tyndarus* (rarely hybridizing), followed by *E. melampus* and *E. sudetica* (hybridization unknown), *E. euryale* and *E. ligea* (hybridization unknown), and finally by the two *E. euryale* subspecies (frequently hybridizing). Photo of *E. ligea* by Yannick Chittaro.

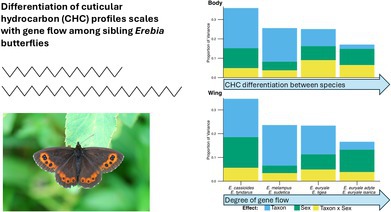

## 1 Introduction

Mountain specialists often harbor genetically unique lineages of significant conservation concern that inhabit spatially limited areas (Sistri et al. 2022). However, their risk of extinction is higher than for many other species due to various factors, including climate‐driven range contractions (Minter et al. 2020; Rödder et al. 2021), phenological shifts (Konvicka et al. 2021), or changes in biotic interactions (Luna and Dáttilo 2024). Climatically induced spatial contact of closely related species may cause maladaptive gene flow between closely related species and the extinction of local endemics (Aitken and Whitlock 2013; Todesco et al. 2016). Closely related species and populations may evolve reproductive isolation mechanisms at different rates, which in many cases prevents hybridization in regions where they coexist (Nice et al. 2013). Consequently, some coexisting sibling species may remain genetically isolated, while others coexist with gene flow (Capblancq et al. 2019; Riesch et al. 2017). Ongoing climatically induced range shifts (Konvicka et al. 2003; Rödder et al. 2021) are likely to lead to secondary contact between previously isolated populations or species in temperate mountain regions with high levels of endemism, such as the European Alps (Dapporto et al. 2024). To successfully predict the potential outcome of such secondary contact, it is crucial to identify potential barriers to gene flow.

Hidden to human perception, differentiation in chemical signaling within and between species may contribute to species divergence by modulating gene flow and hybridization in butterflies (Ômura et al. 2022). Butterfly courtship starts with microhabitat selection (Wilby et al. 2024) and visual stimuli including wing color pattern (Silberglied 1984). Mate identity is further verified via chemical communication, which includes volatile pheromones released by males from androconial scales or other organs, and tactile inspection of cuticular hydrocarbon (CHCs) contact pheromones (Heuskin et al. 2014; Ômura et al. 2020). Sexual selection acts via qualitative variation of specific CHC compounds (Martin and Drijfhout 2009), but also by their quantitative variation (van Zweden et al. 2009). Additionally, some CHCs that are used for communication are released at higher ambient temperatures or after exposure to UV radiation (Blomquist and Ginzel 2021), which is particularly relevant for heliophilous insects such as butterflies. CHCs can evolve rapidly across species phylogenies (Martin and Drijfhout 2009) as well as within species, for example, among populations living under different conditions (Dapporto 2007; Wicker‐Thomas et al. 2015). CHCs synthesis pathways are shared by different groups and are evolutionarily labile due to varied mechanisms that facilitate gene silencing or down regulation (Kather and Martin 2012; Rusuwa et al. 2022) and activation of different transcription factors (Makki et al. 2014). As CHCs primarily have a protective function against desiccation and because they are synthesized in response to climatic conditions (Etges et al. 2017), both environmental (Gibbs et al. 1998; Parkash et al. 2008) and sexual (Steiger and Stökl 2014) selection pressures can influence CHC profile composition. Overall, chemical communication may represent an important yet understudied component for reproductive isolation between species pairs that are closely related, morphologically and ecologically similar, and that cannot coexist completely but often persist in close parapatry with narrow contact zones such as *Erebia* butterflies (Augustijnen et al. 2022; Lucek et al. 2020).

The species‐rich butterfly genus *Erebia* accounts for an iconic part of European Alpine biodiversity and has become a model for studying evolutionary processes (Augustijnen, Bätscher, et al. 2024; Klečková et al. 2023; Schmitt et al. 2016) and conservation genomics (Augustijnen, Patsiou, et al. 2024; Jospin et al. 2023). The drivers underlying the extraordinary diversity of *Erebia* are complex, involving allopatric speciation (Schmitt et al. 2016), variability in chromosome numbers (Augustijnen, Bätscher, et al. 2024) as well as morphological (Cupedo 1996) and ecological (Kleckova et al. 2014; Klečková et al. 2023) differentiations. Postglacial expansion from lowland refugia resulted in a unique pattern of spatial coexistence of closely related species (Schmitt et al. 2016). While many ecologically and morphologically distinct *Erebia* species can coexist over large parts of their ranges (Kleckova et al. 2014), closely related species or subspecies often form narrow zones of secondary contact with only limited gene flow (Augustijnen et al. 2022; Bouaouina et al. 2023), or are allopatric even at close distances (Jospin et al. 2023). However, the evolutionary drivers causing these narrow hybrid zones have remained unclear (Augustijnen et al. 2022; Bouaouina et al. 2023; Sonderegger 2005). *Erebia* is thus an ideal system for the study of reproductive barriers during secondary contact, a topic of increasing importance given the rapid pace of climate change bringing species into contact. The potential for differentiation in CHCs has not been examined in *Erebia*, although divergence in species‐specific mate recognition chemical signals is expected to promote reproductive isolation (Adams and Tsutsui 2020; Engsontia et al. 2014).

Taking advantage of former studies on *Erebia* resolving its phylogeny (Augustijnen, Bätscher, et al. 2024) and mapping secondary contact zones (Augustijnen et al. 2022; Bouaouina et al. 2023; Sonderegger 2005), we chose three *Erebia* sibling species pairs and one pair of sibling subspecies that occur in local sympatry or local parapatry. These fall at different stages along a speciation continuum, that is, from where gene flow is still possible to being completely absent, allowing us to assess whether differentiation in CHCs could be associated with the degree of isolation between taxa. Based on studies on other butterflies (Heuskin et al. 2014) we predict CHC profiles in *Erebia* to differ: (i) between body parts, that is, wings and body, due to their different physiological functions; (ii) between sexes, as this plays an important role during mate choice (Ômura et al. 2012); and (iii) among species pairs, as we predict the level of differentiation in CHCs to scale with the overall degree of differentiation between species pairs.

## 2 Methods

### 2.1 Species and Sampling Sites

**FIGURE 1:**
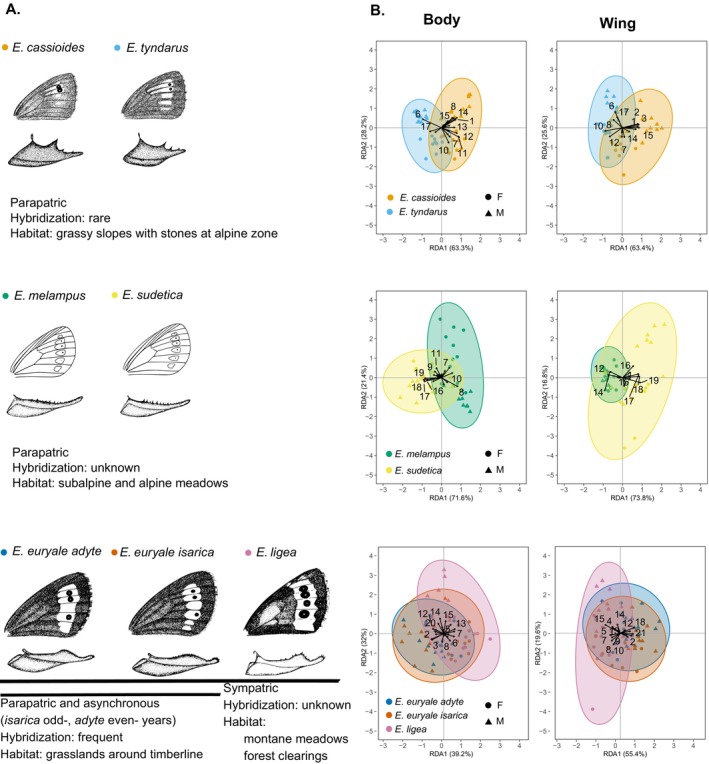
(A) Sibling taxa of *Erebia* butterflies differ in wing patterns and the morphology of male genitalia. Information on habitat and reported hybridization between the species pairs is also indicated. *Erebia tyndarus* and *E. cassioides* differ in the shape of ocelli on the forewing and the size of the middle tooth on valvae. *Erebia sudetica* and *E. melampus* vary in the tone of orange spots, presence of a black dot in the upper orange spot, and the size of the teeth on valvae. *Erebia ligea* males have androconia on their wings and differ from *E. euryale* in the development of black ocelli in the orange band and in the shape of valvae. The subspecies *Erebia euryale adyte* and *E. e. isarica* differ in the presence of white dots in the black ocelli, which are present in *E. e. adyte*, but absent in *E. e. isarica*, as well as in genital morphology. Drawings adapted from Sonderegger (2005) (CC‐BY). (B) Degree of differentiation in the relative abundances of cuticular hydrocarbons (CHCs) reflected the expected strength of reproductive barriers among sibling taxa pairs. Redundancy analysis (RDA) diagrams show variation in the relative abundances of CHCs from body and wings. For *E. euryale*, two subspecies (*E. e. adyte* and *E. e. isarica*) were analysed. CHCs differentiation was assessed between sibling taxa (*E. ligea* and *E. euryale*, including both subspecies), as well as between the two *E. euryale* subspecies (see Table 1).

We collected *Erebia* specimens of three sister species pairs and one subspecies pair (Figure 1A) in the Swiss Alps during summer 2023. The sibling pairs differ in their degree of spatial overlap and gene flow;

1. the two subspecies *Erebia euryale adyte* and *E. e. isarica*, which differ slightly in their wing pattern and morphology of male genitalia (Bouaouina et al. 2023; Figure 1A). The two subspecies are distributed in different parts of the Alps and likely diverged in different glacial refugia (Cupedo and Doorenweerd 2022). The two subspecies form narrow contact zones (Sonderegger 2005), where they overlap spatially. *Erebia e. adyte* flies only in even and *E. e. isarica* primarily in odd years and in lower abundances in even years. Subspecies interbreed in the hybrid zone where they co‐occur together. In the hybrid zone where the subspecies are split by rugged topography, ongoing gene flow is rare (Bouaouina et al. 2023).
2. *Erebia euryale* taken as a whole (including subspecies *adyte* and *isarica*) and the closely related sympatric *E. ligea*. The species differ in wing morphology, male genitalia (Figure 1A), altitudinal range, and habitat preference, with *E. ligea* found in woodland clearings and *E. euryale* around the timber line (Sonderegger 2005). Males of *E. ligea* have androconia, which release volatile pheromones. The two species frequently co‐occur in sympatry across European mountain ranges; however, it is unknown whether they hybridize.
3. the sibling species *Erebia tyndarus* and *E. cassioides* , which are phenotypically differentiated by both wing colour pattern and genital morphology, and form very narrow contact zones where they produce only rarely F1 hybrids (Augustijnen and Lucek 2024).
4. the sibling species *Erebia melampus* and *E. sudetica* . Whereas the former species is widely distributed across Switzerland, the latter only occurs in a small, isolated spot covering a few square kilometres. These two phenotypically very similar species seem to spatially exclude each other, flying only some hundreds of meters apart from each other for at least several decades (Sonderegger 2005). They most likely survived in different glacial refugia and evolved in allopatry (Haubrich and Schmitt 2007); but it is unknown whether interspecific reproductive barriers exist. Admixture between both species might, however, cause genetic diversity loss in *E. sudetica* .

For each sex and species or subspecies, we aimed to collect 8–10 specimens. As *Erebia* are protandric, we generally collected males one to two weeks before the females from the same site (Table S1). Individuals of each species pair were collected from nearby localities to minimize potential artifacts, for example, due to different abiotic conditions during larval development.

### 2.2 Cuticular Hydrocarbons

Following capture, we stored each specimen individually in sterile plastic containers and froze them at −80°C on the same day. For each individual, we separated both pairs of wings (forewings and hindwings) from the body using scissors. Then, one wing pair (left or right forewing and hindwing) and the rest of the body (i.e., head, thorax with legs and abdomen) were each submerged in 0.5 mL n‐hexane in 10 mL gas‐tight glass vials for 10 min. We transferred the extracts into 1.5 mL glass vials for analysis, avoiding a transfer of wing scales. The samples were analysed on a gas chromatography mass spectrometry platform (GC–MS; GC type: 8890A, MS type: 5977B MSD, both Agilent Technologies, Palo Alto, CA, USA), using a HP‐5MS capillary column (30 m × 250 μm × 0.25 μm, Agilent Technologies). Samples were auto‐injected in pulsed splitless mode (inlet at 250°C) at a constant flow rate of Helium at 0.9 mL min^−1^. The following temperature program was used: 45°C held for 3 min, ramping at 22°C min^−1^ to 290°C which was held for 8 min, then 5°C min^−1^ to 310°C. Identification of all measured compounds was carried out through comparison of the obtained mass spectra to those of commercially available standards (C7–C40 saturated alkanes), NIST version 2.3 mass spectral library, and the Kovats retention index library. Peaks were aligned and quantified using MzMine version 3.9.0 (Schmid et al. 2023). Analyses were performed using the relative abundances of measured compounds.

### 2.3 Statistical Analyses

Firstly, we aimed to understand the potential contributions of body parts, sex, and species to the overall variation in CHC composition across all our studied *Erebia*. For this, we performed a Redundancy Analysis (RDA) in the vegan package (Oksanen et al. 2022) using R 4.3.3 (R Core Team 2024). We included standardized and scaled relative CHC compound abundances as response variables and body parts, taxon, sex, and taxon‐to‐sex interaction as explanatory variables. To assess the significance of the fixed effects, we conducted a permutation‐based significance test with 9999 permutations using the anova.cca function of the *vegan* package (Oksanen et al. 2022). We next assessed the degree of CHC differentiation within the three pairs of sibling species and the one subspecies pair. Because the previous analysis showed differences between body and wings, we ran RDAs separately for body and wings and for each of the four species and subspecies pairs, with taxon, sex, and their interaction as fixed effects. We tested the significance of fixed effects by a permutation‐based significance test with 9999 permutations. To quantify the relative importance of sex, taxon, and their interaction in explaining the variance in the CHC composition, we estimated effect sizes as the proportion of total variance of the ANOVA results explained by the fixed effects (taxon, sex, and their interaction).

Finally, to assess differences in the composition and relative abundances of separate CHCs among taxa and sexes, we ran a series of linear models (LMs) for body and wings respectively, followed by ANOVAs. The response variable in each model was the relative abundance of a compound, while the explanatory variables were taxon, sex, and their interaction. For the ANOVAs, we used the type III sums of squares to account for unbalanced designs and to test for main and interaction effects. We first ran LMs for each compound with all species included as explanatory variables to assess whether compounds differed overall among *Erebia* taxa and sexes. Subsequently, we ran LMs for each compound and taxon pair, using taxon, sex, and their interaction as explanatory variables. To account for multiple testing, we applied a Benjamini and Yekutieli (2001) correction to *p*‐values obtained from the individual ANOVAs.

## 3 Results

**FIGURE 2:**
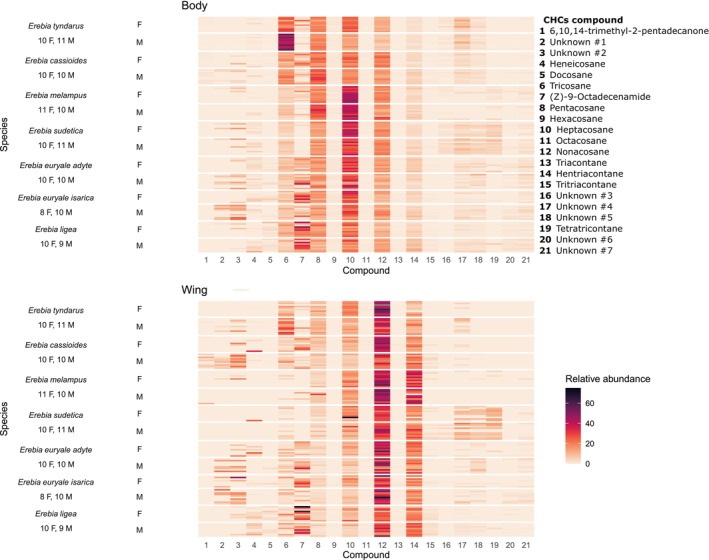
Heat map of relative abundances of the 21 identified cuticular hydrocarbons (CHCs, see Table S2 for tentative compound identification) on body (top) and wing (bottom) body parts for males (M) and females (F) of six *Erebia* butterfly species. *Erebia euryale* was represented by two subspecies, *E. e. adyte* and *E. e. isarica*. Compounds are arranged in ascending order based on their retention indices. Each line represents an individual; sample sizes are indicated below the species name. The colour intensity is proportional to the relative abundance of each compound.

We sampled a total of 140 individuals from seven taxa and detected 21 compounds (Figure 2, Table S2), tentatively identifying 12 alkanes, two long chain hydrocarbons, and fatty acid derivatives, while seven compounds remained unidentified (Table S2). While some compounds were shared among species, others differed. For instance, *Erebia cassioides* and *E. tyndarus* did not include tetratricontane (Tables S3 and S4) or *E. euryale* and *E. ligea* were specific by the absence of the unknown compound #3.

### 3.1 Overall CHC Profile Differences Among Species, Sex, and Body Parts

The redundancy analysis (RDA) including all species showed that the CHC composition differed between *Erebia* taxa (explained relative variance from total variance = 4.85, *F*_6,265_ = 19.13, *p* < 0.001), body part (variance = 3.47, *F*_1,265_ = 82.08, *p* < 0.001) and sex (variance = 0.42, *F*_1,265_ = 9.97, *p* < 0.001). This suggests higher variation in CHCs between body parts than between sexes. The interaction between taxon and sex was also significant (variance = 1.04, *F*_6,265_ = 4.10, *p* < 0.001) suggesting that intersexual differences vary between taxa. Body and wings clustered into separate groups along the first RDA axis, accounting for 37.1% of the overall variance (Figure S1). The second RDA axis, accounting for 20.6% of the overall variation, primarily separated by species.

### 3.2 Differentiation in CHC Profiles Within Sibling Taxa Pairs

Using multivariate analyses, we found significant interspecific differences in relative CHC abundances on both body and wings for all but one taxon pair (*E. e. adyte* and *E. e. isarica*; Table 1, Figure 1B). We further identified significant sexual dimorphisms in the relative abundances of CHC compounds on both body and wings in all species pairs, except for *E. melampus* and *E. sudetica* , for which sexual dimorphisms occurred only on the body, but not on the wings. The species‐to‐sex interaction was significant for both body parts for *E. cassioides* and *E. tyndarus*, as well as for *E. euryale* and *E. ligea*. For *E. melampus* and *E. sudetica* , the interaction between species and sex was only close to significance (*p* = 0.047) for the body but non‐significant for the wings, whereas for the two *E. euryale* subspecies the interaction was only significant for the body. The clustering in the RDA differed among taxon pairs and was the highest for *E. cassioides* and *E. tyndarus* and lowest for the two *E. euryale* subspecies (Figure 1B). The first RDA axis separated species, particularly for body but for some also for wings (i.e., *E. cassioides* and *E. tyndarus*, *E. euryale* and *E. ligea*). The second RDA axis separated males and females for *E. melampus* and *E. sudetica* body (but not wings) and for *E. cassioides* and *E. tyndarus* body and wings.

**TABLE 1:** Results of permutation tests for redundancy analyses (RDAs) comparing related sibling pairs of butterflies in the genus *Erebia* and the two subspecies of *E. euryale*.

Taxon pair	Variable	Body				Wing			
		DF	Variance	*F*	*p*	DF	Variance	*F*	*p*
*E. cassioides—E. tyndarus*	Taxon	1	4.16	12.00	≤ 0.001	1	2.58	9.14	≤ 0.001
	Sex	1	2.06	5.94	≤ 0.001	1	2.07	7.32	≤ 0.001
	Taxon × Sex	1	0.98	2.82	0.007	1	0.90	3.20	0.002
	Residual	37	12.81			37	10.45		
*E. sudetica—E. melampus*	Taxon	1	3.46	8.84	≤ 0.001	1	2.73	8.50	≤ 0.001
	Sex	1	0.90	2.31	0.022	1	0.51	1.57	0.092
	Taxon × Sex	1	0.75	1.91	0.047	1	0.54	1.69	0.069
	Residual	38	14.88			38	12.22		
*E. euryale—E. ligea*	Taxon	2	1.76	3.00	≤ 0.001	2	2.29	4.02	≤ 0.001
	Sex	1	1.45	4.93	≤ 0.001	1	1.22	4.28	≤ 0.001
	Taxon × Sex	2	1.81	3.07	≤ 0.001	2	0.93	1.63	0.041
	Residual	51	15			51	14.6		
*E. eu. adyte—E. eu. issarica*	Taxon	1	0.42	0.90	0.532	1	0.63	1.36	0.163
	Sex	1	1.60	3.44	≤ 0.001	1	1.79	3.84	≤ 0.001
	Taxon × Sex	1	1.23	2.65	0.006	1	0.73	1.57	0.092
	Residual	34	15.76			34	15.85		

*Note:* While the closely related species pairs of *Erebia* butterflies differentiated in their chemical profiles, the subspecies of *E. euryale (adyte* and *isarica*) did not differ. Differentiation between sexes was low in *E. melampus* and *E. sudetica* .

**FIGURE 3:**
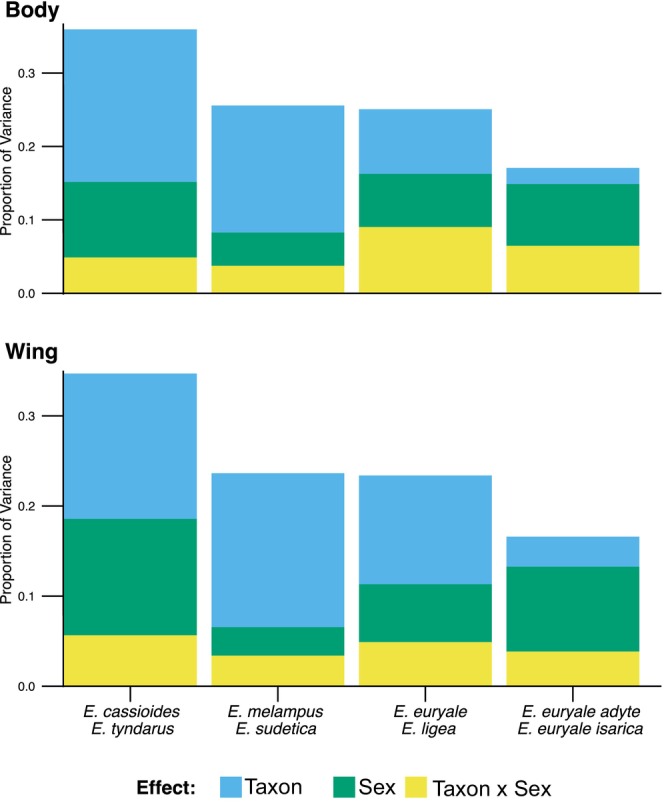
Effect sizes of variance explained by redundancy analyses (RDAs) comparing cuticular hydrocarbon profiles of related species and subspecies pairs of *Erebia* butterflies. The effect size represents the proportion of total variance explained by taxon, sex and their interaction. Variability attributed to taxon was highest in *E. cassioides* and *E. tyndarus*, followed by *E. melampus* and *E. sudetica* , *E. euryale* and *E. ligea,* and finally by the two *E. euryale* subspecies. The low proportion of explained variability by sex and taxon‐to‐sex related differences in *E. melampus* and *E. sudetica* suggests a lack of compounds participating in mate recognition in these species.

The effect sizes of variance explained by taxon, sex, and taxon‐to‐sex interaction in the RDA confirmed a gradient of differentiation of CHC profiles between taxa (Figure 3). The strongest interspecific differentiation of CHCs was revealed in body and wings between *E. tyndarus* and *E. cassioides*, and between *E. melampus* and *E. sudetica*. In these two species pairs, the variation explained by interspecific differences was higher than the variation explained by sex. In *E. euryale* and *E. ligea*, the species effect was comparable to the effect of sex. For the *E. euryale* subspecies, sex‐specific differences explained more variation in CHC relative abundances compared to subspecies‐specific differences. The interaction between sex and taxon accounted for the least amount of variation in all species pairs and was the lowest in *E. melampus* and *E. sudetica* (body and wings) and in wings of *E. euryale* and *E. ligea*.

### 3.3 Individual Compound Differences Between Sibling Taxa Pairs

Interspecific differences in relative abundances of the CHC compounds were distinct between the three species pairs (Table S4): *E. cassiodes* and *E. tyndarus* differed in relative abundances for nine compounds on the body (Figure S2) and for eight compounds on the wings (Figure S3); *E. melampus* and *E. sudetica* differed for five compounds on the body (Figure S4) and five on the wings (Figure S5) and *E. ligea* and *E. euryale* for one compound on the body (Figure S6) and two compounds on the wings (Figure S7). Compound abundances did not differ between the *E. euryale* subspecies (Table S4, Figures S6 and S7). The sex‐specific differences in compound abundances were distinct in all taxon pairs (Table S4) except for *E. melampus* and *E. sudetica* . Males and females differed for five compounds on the body and seven compounds on the wings in *E. cassiodes* and *E. tyndarus*, for one compound on the body in *E. melampus* and *E. sudetica* , for one compound on the body and two compounds on the wings in *E. ligea* and *E. euryale*, and for two compounds on the wings in the *E. euryale* subspecies. The interactive effect of taxon and sex in relative compound abundances was significant for four compounds on the body and for three compounds on the wings between *E. cassiodes* and *E. tyndarus*, for two compounds on the body between *E. euryale* and *E. ligea* but not between *E. melampus* and *E. sudetica* or *E. euryale* subspecies (Table S4). The observed differentiation in individual compounds is congruent with overall multivariate differentiation in the RDA analyses (Table 1). However, the absence of significant sex and taxon‐to‐sex interactions in the *E. melampus* and *E. sudetica* pair and *E. euryale* subspecies is not congruent with the overall RDA analyses (Table 1), which found marginal significances of the sex and the taxon‐to‐sex interaction in the body in *E. melampus* and *E. sudetica* and in the body in *E. euryale* subspecies.

## 4 Discussion

We observed a gradient in the differentiation of cuticular hydrocarbons (CHCs) among four pairs of closely related *Erebia* species or subspecies (Table S4, Figures 1 and 3), which vary in their degree of gene flow and local overlap of distribution (Sonderegger 2005; Augustijnen and Lucek 2024; Bouaouina et al. 2023; Figure 1). The extent of interspecific and sex‐specific CHC differentiation (Table S4) may on the one hand reflect differentiation as a by‐product of phases of allopatry, but on the other hand be also important for chemical communication in maintaining reproductive barriers between closely related taxa. Differentiation in CHCs was previously hypothesized to contribute to reproductive isolation between sympatric arthropods, including *Drosophila* flies (Fezza et al. 2022), stick insects (Schwander et al. 2013) and grasshopper subspecies (Neems and Butlin 1997). However, differentiation in CHC profiles may not always be involved in reproductive isolation, as has been shown for *Coccinella* beetles (Redjdal et al. 2023). The importance of CHC differentiation as a reproductive barrier therefore likely varies among taxa (Buellesbach et al. 2013) and populations (Buckley et al. 2003). In butterflies, some CHCs are thought to be under sexual selection (Dapporto 2007), functioning as contact pheromones during mate choice (see Heuskin et al. 2014; Ômura and Yotsuzuka 2015). Former studies on *Erebia* have speculated on which degree CHCs between sibling species are distinct (Augustijnen et al. 2022).

The most differentiated CHC profiles for both species and sexes were between locally parapatric *E. cassioides* and *E. tyndarus* (Figures 1 and 3; Table S4). The two species only rarely hybridize upon secondary contact, with almost no evidence for contemporary gene flow (Augustijnen and Lucek 2024). Interspecific and sex‐specific patterns of CHCs were also distinct between locally sympatric *E. euryale* and *E. ligea*, but to a lower extent than between *E. cassioides* and *E. tyndarus*. This reduced differentiation in *E. euryale* and *E. ligea* probably relates to other barriers to gene flow. Although the two species frequently co‐occur across their range, they differ in climatic niches (Klečková et al. 2023), microhabitat use (Kleckova et al. 2014) and in the presence of androconia in *E. ligea*, which are absent in *E. euryale* (Sonderegger 2005). Volatile compounds emitted from androconia represent primary chemical mating cues but were unrecognised by our study. In both pairs, significant interactions between species and sex explained variation of CHC profiles (Table 1) and differences in relative abundances of several CHC compounds (Table S4). Especially, some of the compounds with recognised significant species‐to‐sex interactions (Table S4) might participate in chemical communication and could act as a prezygotic barrier (Fezza et al. 2022) in these pairs; yet formal experiments are needed.

The CHC profiles of the remaining two pairs are differentiated between taxa or sexes, but to a lesser extent than in the aforementioned species pairs. Locally parapatric *E. melampus* and *E. sudetica* , which probably have not come into spatial contact since their recolonization of the Alps (Sonderegger 2005), differ in their chemical profiles (Table 1, Table S4). However, differentiation between males and females is comparatively low as indicated by low overall CHCs differences (Table 1, Figures 1 and 3), which differed in relative abundances of pentacosane for body (Figure S4) but not wings (Figure S5, Tables S3 and S4). Hybridization between the highly protected and isolated *E. sudetica* populations with the more widespread *E. melampus* could result in maladaptive gene flow and potentially lead to the local extinction of *E. sudetica* .

The CHC profiles of the two *E. euryale* subspecies did not differ (Tables S1 and S4) despite their morphological differentiation (Sonderegger 2005; Figure 1). Consistently, the two subspecies of *E. euryale* show a relatively high level of genome‐wide genetic differentiation between allopatric sites, but both subspecies hybridize at their contact zones (Bouaouina et al. 2023). Taken together, we found different levels of differentiation in CHCs that seem to scale with the degree of apparent reproductive isolation between our species pairs (Augustijnen and Lucek 2024; Bouaouina et al. 2023; Haubrich and Schmitt 2007), suggesting that CHCs could be directly or indirectly involved in species and mate recognition. If CHCs have a signalling function, diversification of CHCs across *Erebia* species should be tightly linked to the evolution of olfactory genes (Torres‐Oliva et al. 2016). Indeed, diversification in olfactory genes contributes to butterfly speciation (Carlsson et al. 2013; Wu et al. 2022), but our understanding of the genes encoding CHCs and the regulation of their expression yet remains limited (Moris et al. 2023), especially in *Erebia*. Phylogenetic inferences suggest that CHCs derived from the same biosynthetic pathways evolve more rapidly than biosynthetic pathways associated with different CHC classes (Menzel et al. 2017). The identified alkanes across all *Erebia* species (Table S2) are synthesised through a similar pathway, suggesting that their evolution could be relatively flexible. Furthermore, alkane composition can be altered through transcriptomic changes and by modification of CHC synthesis pathways (Martin and Drijfhout 2009; Menzel et al. 2017). The future availability of *Erebia* chromosome‐resolved genomes (Mazzoni et al. 2023) and the ease with which transcriptomes can be generated for individuals experiencing different climates (e.g., along elevation gradients) offer promising opportunities to identify genes responsible for CHC synthesis (Blomquist and Ginzel 2021; Etges et al. 2017; Holze et al. 2021). To assess the potential for intraspecific variation among populations, future research should compare multiple populations (Neems and Butlin 1997) and/or assess chemical profile changes based on distance from hybrid zones of parapatric species, allowing us to infer whether reinforcing selection could act on them.

Although it is unknown whether and to which degree CHCs might be involved in mate choice in *Erebia*, some of our tentatively identified CHCs (Table S2) have been shown to be involved during courtship in other butterfly species. Specifically, heneicosane, tricosane, pentacosane, heptacosane, nonacosane, and triacontane involved in interspecific and sex‐specific recognition as confirmed by electroantennographic experiments in other Nymphalids (Ehlers and Schulz 2023). Such CHCs have also been identified as mating signals in other insects: 6,10,14‐trimethyl‐2‐pentadecanone is involved in mate recognition of click beetles (Singleton et al. 2022), tricosane attracts females of a moth species (Grant et al. 1987), pentacosane is a contact pheromone of a weevil (Sun et al. 2017), and heptacosane was revealed to attract males in *Colias* butterflies (Grula et al. 1980). These studies, however, often involved lab‐reared individuals and mate choice tests, which were beyond the scope of our study. *Erebia* have frequently prolonged development, taking up to two years, and they are challenging to breed in captivity (Heuskin et al. 2014). Thus, the possible effects of age or conditions experienced during development on CHC differentiation remain to be tested. While the often strong differentiation in CHC profiles between the body and the wings (Table 1, Figures S1 and S2) could reflect specific physiological functions of the body parts, it might also correspond with mate signaling during courtships (Grula et al. 1980). In *Erebia*, females sit on grass to attract patrolling males by making specific movements (Brussard and Ehrlich 1970). When a male lands close to a female, he touches the body of the female with his antennae and walks around her, flapping his wings (Kuras et al. 2001). During this phase, *Erebia* may explore CHCs of different body parts of their mates; behavioral observation on *Erebia* confirmed that males are attracted to pinned, dead females (Brussard and Ehrlich 1970). Still, behavioral assessments, such as mate choice experiments with dummy models or electroantennography, are needed to confirm the role of CHCs in sexual signaling (Slone et al. 2017).

Tactile inspection of CHCs might further interact with thermoregulation. Temperature changes may cause changes in CHCs leading to their degradation to volatile chemicals that can also have signaling function (Blomquist and Ginzel 2021). *Erebia* maintain specific body temperatures by searching for optimal microclimates—rocky habitat species ( *E. cassioides* , *E. tyndarus*) maintain warmer body temperatures than grassland ( *E. sudetica* , *E. melampus*) and woodland (*E. euryale*, E. *ligea*) species (Kleckova et al. 2014). The maintenance of higher body temperatures for *E. cassioides* and *E. tyndarus*, combined with their wings with long, easily releasable scales, is consistent with a potential role of scales in chemical advertisement of species identity.

## 5 Conclusions

Our study provides the first report of cuticular hydrocarbons (CHCs) differentiation among sympatric and locally parapatric *Erebia* butterflies. The gradient of differentiation in CHCs among *Erebia* species pairs seems to scale with their level of reproductive isolation. While this differentiation could result from random genetic drift or local adaptation associated with species divergence in allopatry, it could also be linked to species and mate recognition. In the latter case, CHC‐mediated chemical communication could contribute to reproductive isolation, supporting species differentiation and long‐term maintenance following secondary contact due to climate‐ induced range shifts in temperate mountains. Differentiation in CHCs and olfactory receptors may help individuals to recognize conspecific mates among previously isolated allopatric lineages and could act as reproductive barriers even at an early stage of speciation, that is, before noticeable morphological differences emerge. At the same time, CHCs divergence could also reinforce species boundaries during secondary contact by limiting interspecific gene flow (Hood et al. 2022). The respective contributions of CHCs in mate choice and species differentiation, especially during secondary contact, require further experimental studies. Comparative analyses across many more sister species pairs with varying divergence times could similarly reveal whether and to which degree species diversification could be associated with CHC differentiation. Taken together, with climate change affecting alpine species' ranges, understanding cryptic CHC diversity and their potential for reproductive isolation is vital for biodiversity management in these ecosystems.

## Author Contributions

**Irena Kleckova:** conceptualization (equal), data curation (equal), formal analysis (equal), funding acquisition (equal), investigation (equal), methodology (equal), resources (equal), visualization (equal), writing – original draft (lead). **Mary Veronica Clancy:** data curation (equal), formal analysis (equal), methodology (equal), software (equal), writing – review and editing (equal). **Camille Cornet:** conceptualization (equal), formal analysis (equal), investigation (equal), methodology (equal), visualization (equal), writing – review and editing (equal). **Nadir Alvarez:** funding acquisition (equal), investigation (equal), resources (equal), writing – review and editing (equal). **Pável Matos‐Maraví:** investigation (equal), resources (equal), writing – review and editing (equal). **Kay Lucek:** conceptualization (equal), formal analysis (equal), investigation (equal), methodology (equal), resources (equal), supervision (lead), writing – review and editing (equal).

## Conflicts of Interest

The authors declare no conflicts of interest.

## Supporting information


**Figure S1:** Redundancy analyses (RDAs) comparing relative abundances of cuticular hydrocarbon (CHCs) compounds occurring on body and wings of related *Erebia* butterfly taxa. The first RDA axis separated wings and body. The second RDA axis corresponds to taxa identity.

**Figure S2:** The relative abundances of individual cuticular hydrocarbons on the body of two sibling species, *Erebia cassioides* and *E. tyndarus*. These species occur in parapatry and rarely hybridize in secondary contact zones.

**Figure S3:** The relative abundances of individual cuticular hydrocarbons on wings of two sibling species, *Erebia cassioides* and *E. tyndarus*. These species occur in parapatry and rarely hybridize in secondary contact zones.

**Figure S4:** The relative abundances of individual cuticular hydrocarbons on body of two sibling species, *Erebia melampus* and *E. sudetica* . These species occur in parapatry—they spatially exclude each other, flying several hundreds of meters apart from each other for at least several decades. It is not known whether the species hybridize.

**Figure S5:** The relative abundances of individual cuticular hydrocarbons on wings of two sibling species, *Erebia melampus* and *E. sudetica* . These species occur in parapatry—they spatially exclude each other, flying several hundred meters apart from each other for at least several decades. It is not known whether the species hybridize.

**Figure S6:** The relative abundances of individual cuticular hydrocarbons on body of two sibling species, *Erebia euryale* and *E. ligea*. *Erebia euryale* consists of two subspecies, *E. e. adyte* and *E. e. isarica*, which differ slightly in their wing patterns and the morphology of the male genitalia. *Erebia euryale* and *E. ligea* co‐occur in local sympatry across their range, and it is not known whether they hybridize. The *E. euryale* subspecies are parapatric and frequently hybridize in zones of secondary contact.

**Figure S7:** The relative abundances of individual cuticular hydrocarbons on wings of two sibling species, *Erebia euryale* and *Erebia ligea*. *Erebia euryale* consists of two subspecies, *E. e. adyte* and *E. e. isarica*, which differ slightly in their wing patterns and the morphology of the male genitalia. *Erebia euryale* and *E. ligea* co‐occur in local sympatry across their range, and it is not known whether they hybridize. The E. *euryale* subspecies are parapatric and frequently hybridize in zones of secondary contact.

**Table S1:** Individual identity, date and site of collection, and body part from which cuticular hydrocarbon compounds (CHCs) were extracted and corresponding relative abundances of tentatively identified CHCs.

**Table S2:** Tentatively identified cuticular hydrocarbon (CHCs) compounds arranged by their retention index. Compounds verified by authentic standard are underlined.

**Table S3:** Average values of relative abundances of tentatively identified cuticular hydrocarbon compounds (CHCs; Table S2) in females (F) and males (M) in four sibling taxa pairs of *Erebia* butterflies.

**Table S4:** Differences in relative abundances of each identified compound (Table S2, Figures S2–, S7) among all *Erebia* butterfly sibling taxa pairs. The linear models for each compound with relative abundance of a CHC compound as response variable and taxon, sex and taxon‐to‐sex interaction as explanatory variables, individual identity was included as fixed effect. Significance of the dependence was tested by ANOVAs. To account for multiple testing, we applied a Benjamini‐Yekutieli correction.

## Data Availability

All data and scripts are archived on Figshare (https://doi.org/10.6084/m9.figshare.28070693.v1).
