# Supplementary figures and images for: Co‐Occurring Sister Taxa of Mountain Butterflies Exhibit Distinct Cuticular Hydrocarbon Profiles
Source: Ecol Evol. 2025 Sep 3;15(9):e72027. doi: 10.1002/ece3.72027 (PMC12405801; doi:10.1002/ece3.72027)

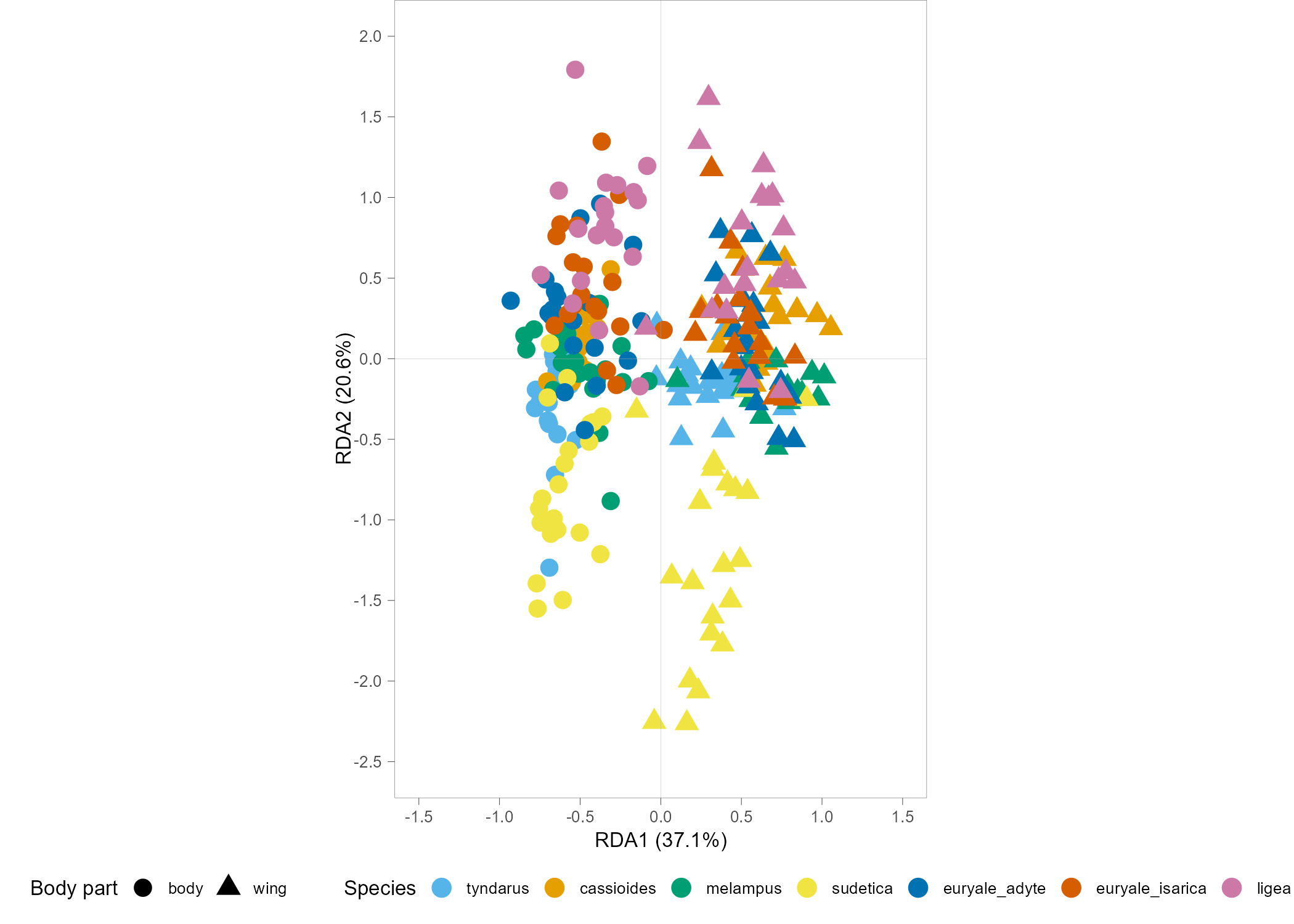

Supplement: Supplementary file 1 — Figure S1: Redundancy analyses (RDAs) comparing relative abundances of cuticular hydrocarbon (CHCs) compounds occurring on body and wings of related Erebia butterfly taxa. The first RDA axis separated wings and body. The second RDA axis corresponds to taxa identity. [file ECE3-15-e72027-s007.tiff]

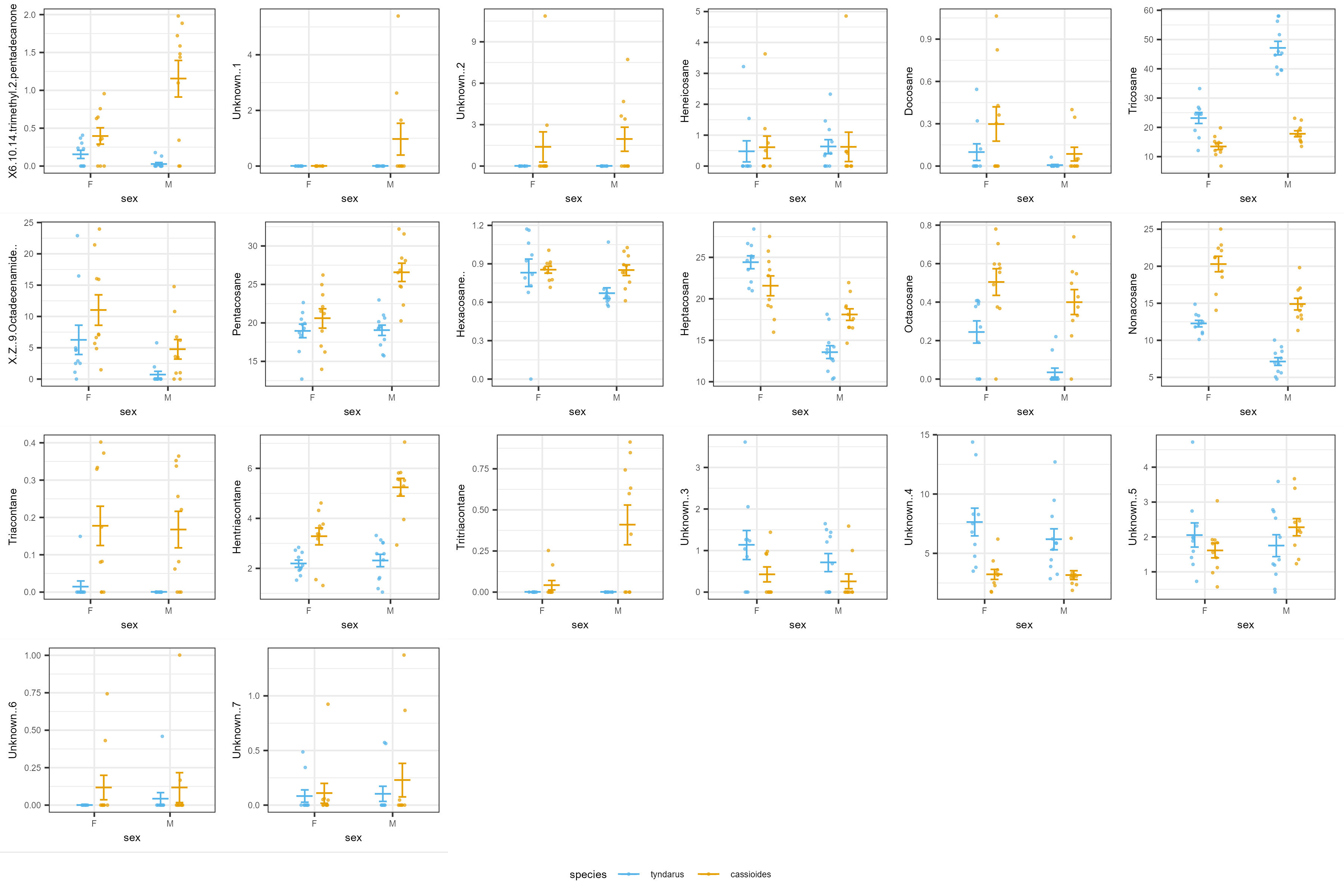

Supplement: Supplementary file 2 — Figure S2: The relative abundances of individual cuticular hydrocarbons on the body of two sibling species, Erebia cassioides and E. tyndarus. These species occur in parapatry and rarely hybridize in secondary contact zones. [file ECE3-15-e72027-s005.tiff]

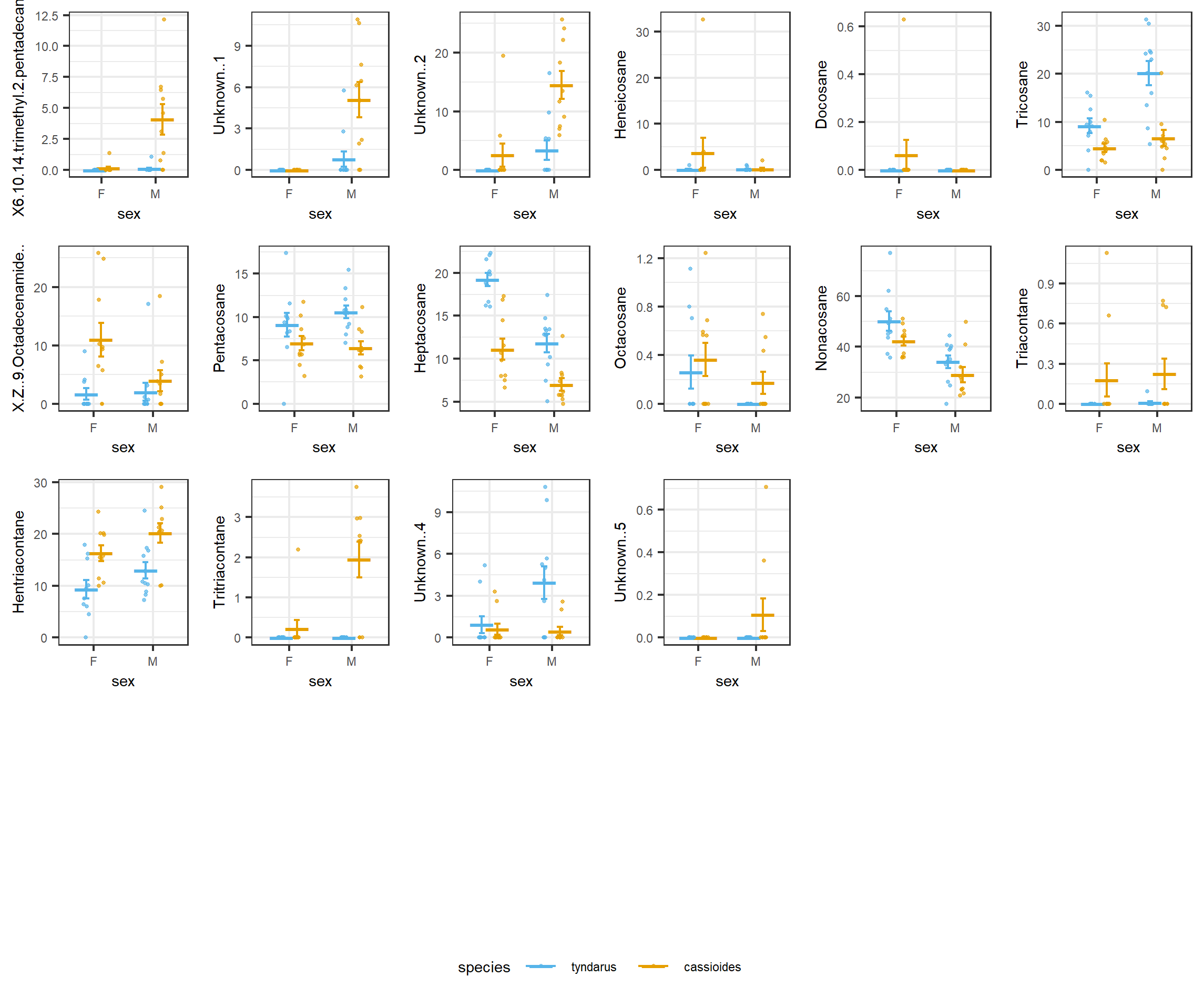

Supplement: Supplementary file 3 — Figure S3: The relative abundances of individual cuticular hydrocarbons on wings of two sibling species, Erebia cassioides and E. tyndarus. These species occur in parapatry and rarely hybridize in secondary contact zones. [file ECE3-15-e72027-s003.tiff]

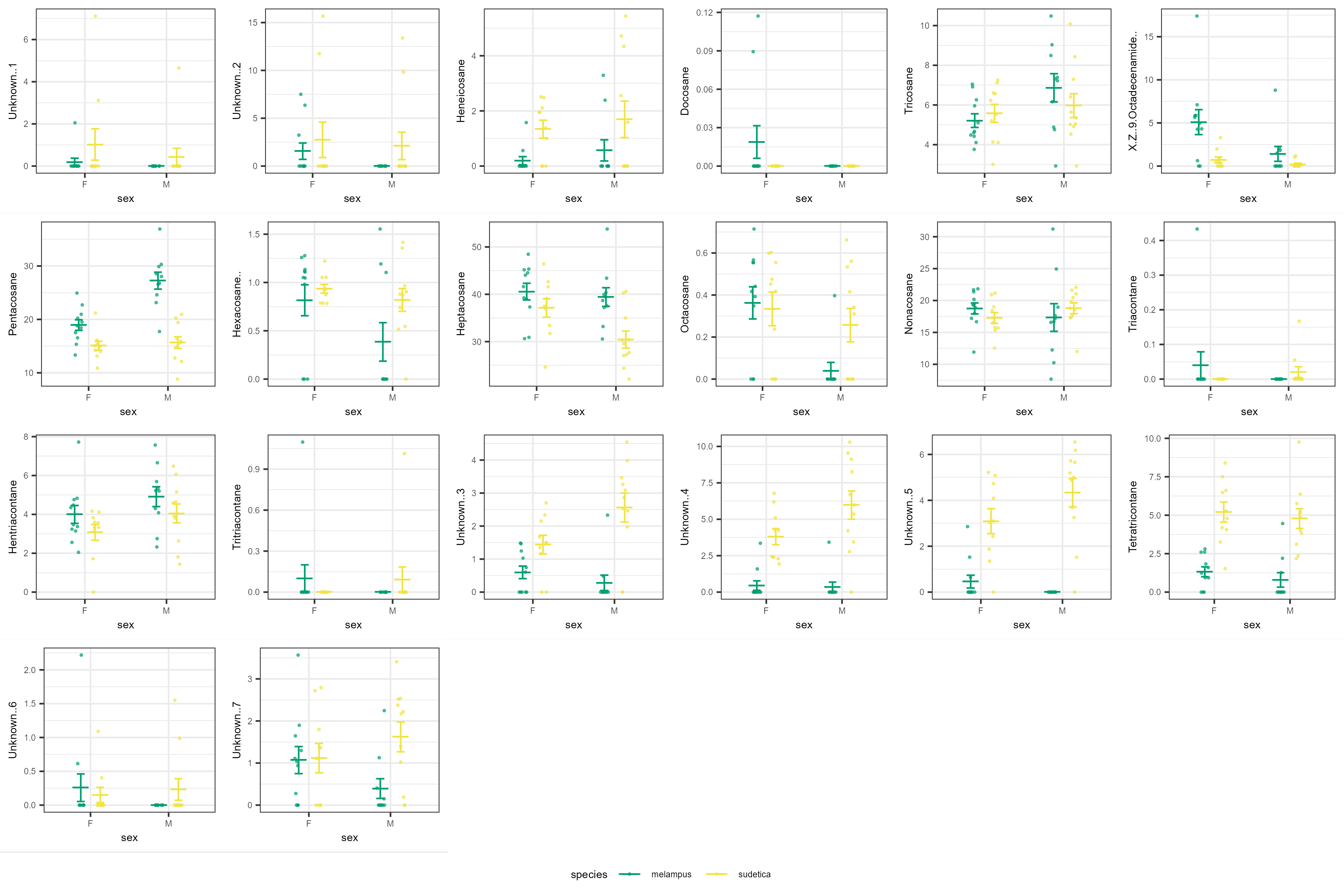

Supplement: Supplementary file 4 — Figure S4: The relative abundances of individual cuticular hydrocarbons on body of two sibling species, Erebia melampus and E. sudetica . These species occur in parapatry—they spatially exclude each other, flying several hundreds of meters apart from each other for at least several decades. It is not known whether the species hybridize. [file ECE3-15-e72027-s009.tiff]

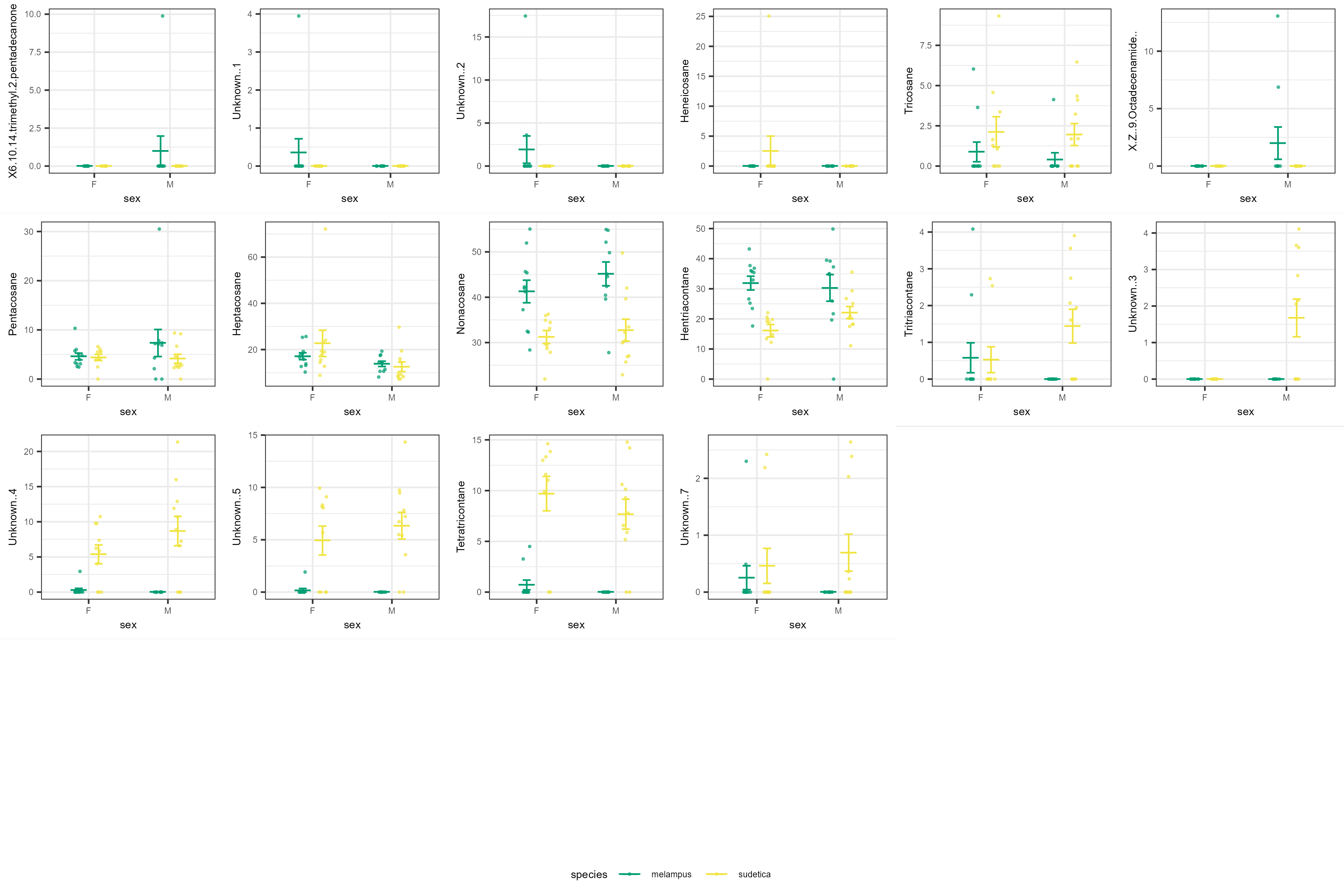

Supplement: Supplementary file 5 — Figure S5: The relative abundances of individual cuticular hydrocarbons on wings of two sibling species, Erebia melampus and E. sudetica . These species occur in parapatry—they spatially exclude each other, flying several hundred meters apart from each other for at least several decades. It is not known whether the species hybridize. [file ECE3-15-e72027-s011.tiff]

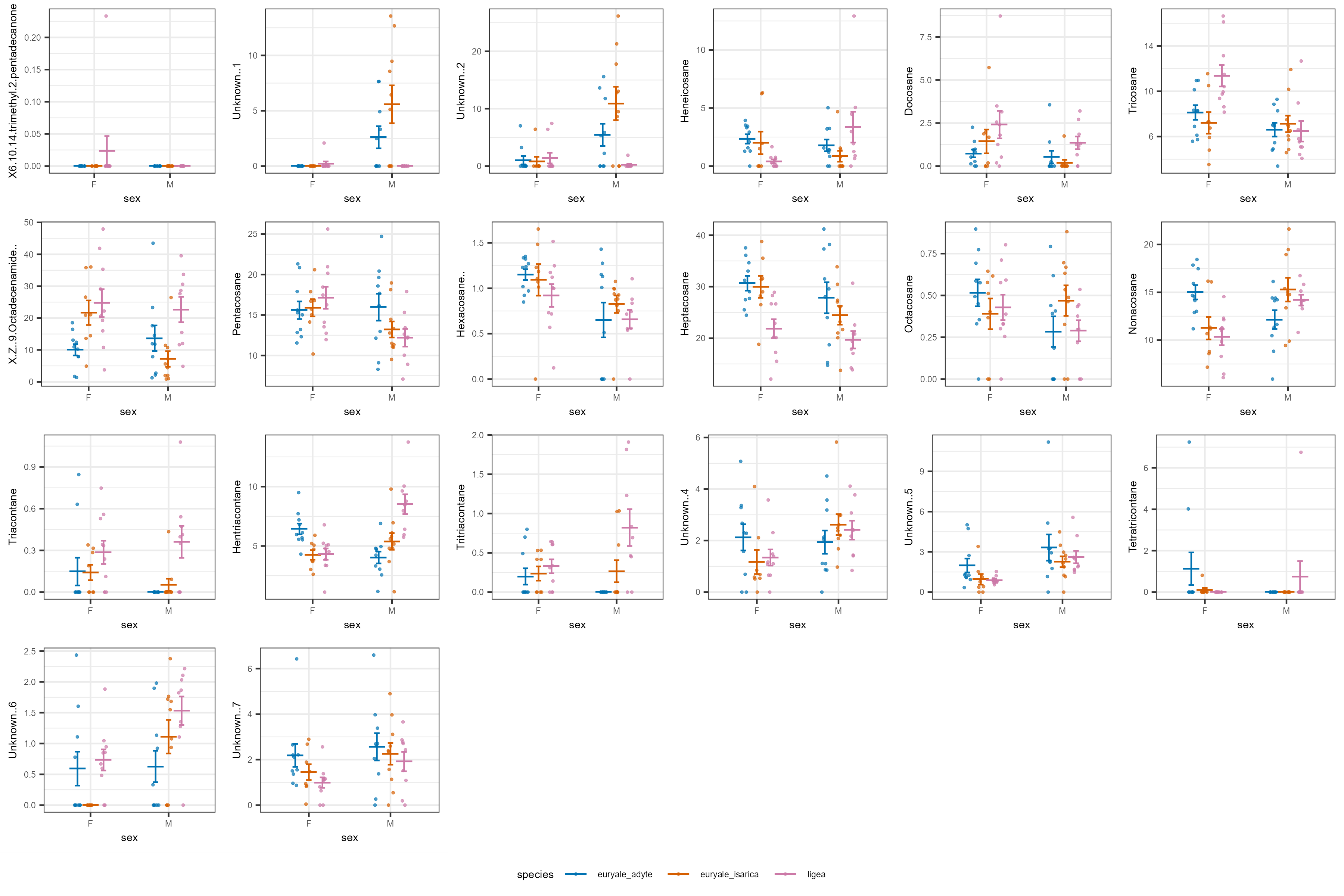

Supplement: Supplementary file 6 — Figure S6: The relative abundances of individual cuticular hydrocarbons on body of two sibling species, Erebia euryale and E. ligea. Erebia euryale consists of two subspecies, E. e. adyte and E. e. isarica, which differ slightly in their wing patterns and the morphology of the male genitalia. Erebia euryale and E. ligea co‐occur in local sympatry across their range, and it is not known whether they hybridize. The E. euryale subspecies are parapatric and frequently hybridize in zones of secondary contact. [file ECE3-15-e72027-s001.tiff]

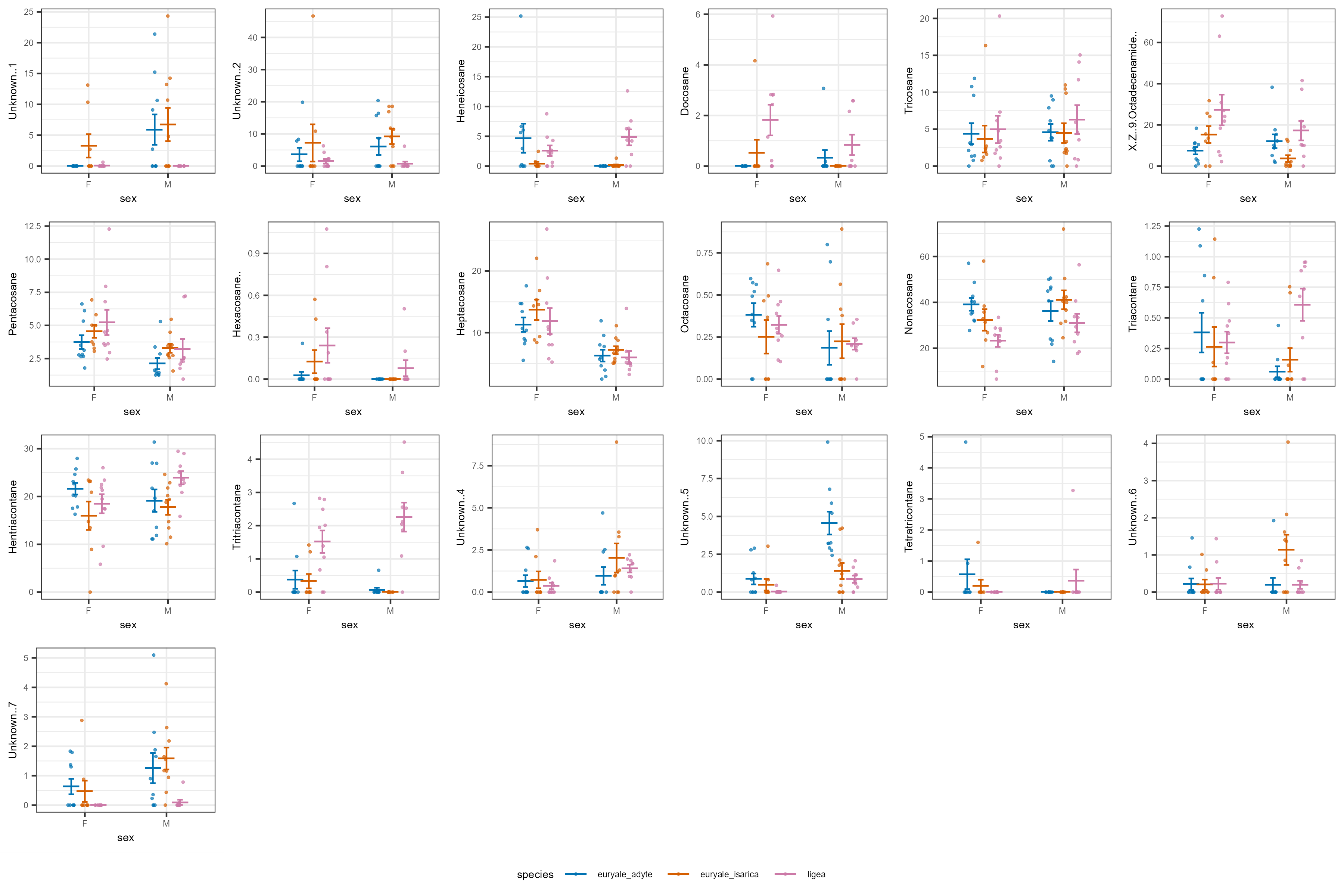

Supplement: Supplementary file 7 — Figure S7: The relative abundances of individual cuticular hydrocarbons on wings of two sibling species, Erebia euryale and Erebia ligea. Erebia euryale consists of two subspecies, E. e. adyte and E. e. isarica, which differ slightly in their wing patterns and the morphology of the male genitalia. Erebia euryale and E. ligea co‐occur in local sympatry across their range, and it is not known whether they hybridize. The E. euryale subspecies are parapatric and frequently hybridize in zones of secondary contact. [file ECE3-15-e72027-s002.tiff]
